# *owlcpp*: a C++ library for working with OWL ontologies

**DOI:** 10.1186/s13326-015-0035-z

**Published:** 2015-09-16

**Authors:** Mikhail K. Levin, Lindsay G. Cowell

**Affiliations:** Department of Clinical Sciences, University of Texas Southwestern Medical Center, 5323 Harry Hines Boulevard, Dallas, TX USA

## Abstract

**Background:**

The increasing use of ontologies highlights the need for a library for working with ontologies that is efficient, accessible from various programming languages, and compatible with common computational platforms.

**Results:**

We developed *owlcpp*, a library for storing and searching RDF triples, parsing RDF/XML documents, converting triples into OWL axioms, and reasoning. The library is written in ISO-compliant C++ to facilitate efficiency, portability, and accessibility from other programming languages. Internally, *owlcpp* uses the Raptor RDF Syntax library for parsing RDF/XML and the FaCT++ library for reasoning. The current version of *owlcpp* is supported under Linux, OSX, and Windows platforms and provides an API for Python.

**Conclusions:**

The results of our evaluation show that, compared to other commonly used libraries, *owlcpp* is significantly more efficient in terms of memory usage and searching RDF triple stores. *owlcpp* performs strict parsing and detects errors ignored by other libraries, thus reducing the possibility of incorrect semantic interpretation of ontologies. *owlcpp* is available at http://owl-cpp.sf.net/ under the Boost Software License, Version 1.0.

**Electronic supplementary material:**

The online version of this article (doi:10.1186/s13326-015-0035-z) contains supplementary material, which is available to authorized users.

## Background

Ontologies are being increasingly recognized as important information resources that capture descriptive information in a standardized, structured, and computable form. One of the most widely used approaches for representing ontologies is the family of languages referred to as the Web Ontology Language (OWL) [1]. The OWL languages were designed to represent ontologies for use in the Semantic Web and were therefore built on the W3C semantic web stack, which includes XML, XML Schema, RDF, and RDF Schema [2–5].

Working with OWL ontologies involves several common procedures, including parsing ontology documents, storing them as RDF triples and axioms, querying and serializing their in-memory representation, passing the axioms to a reasoner, and performing logical queries. Given the increasing size of ontologies, it is extremely important to have software for working with OWL ontologies that can perform these procedures efficiently.

During the last decade, many open-source libraries useful for working with OWL ontologies written in RDF+XML format have become available. These, however, fail to fully meet the needs of software developers building software for working with OWL ontologies. First, existing libraries do not scale well enough to support ontologies of over a few million triples [6, 7]. In addition, the majority of them are implemented in non-native languages, which are usually less efficient and involve significant overhead when accessed from other languages [8]. For example, the libraries with the most extensive functionality, OWL API [9, 10] and Apache Jena [11, 12], are implemented in Java. Given the difficulties of accessing Java from other languages, it is not surprising that the recent Perl and Python libraries ONTO-PERL [13], RDFLIB [14], and FuXi [15], replicate some of the functionality already present in OWL API and Jena. Redland RDF framework is implemented in C and provides utilities for parsing, storing, and querying RDF triples [16, 17]. Its native code base allows it to more easily expose its API in several other languages and to be usable on virtually any platform, including mobile devices [18]. The functionality of Redland is limited, though, because it does not directly support OWL.

**Fig. 1:**
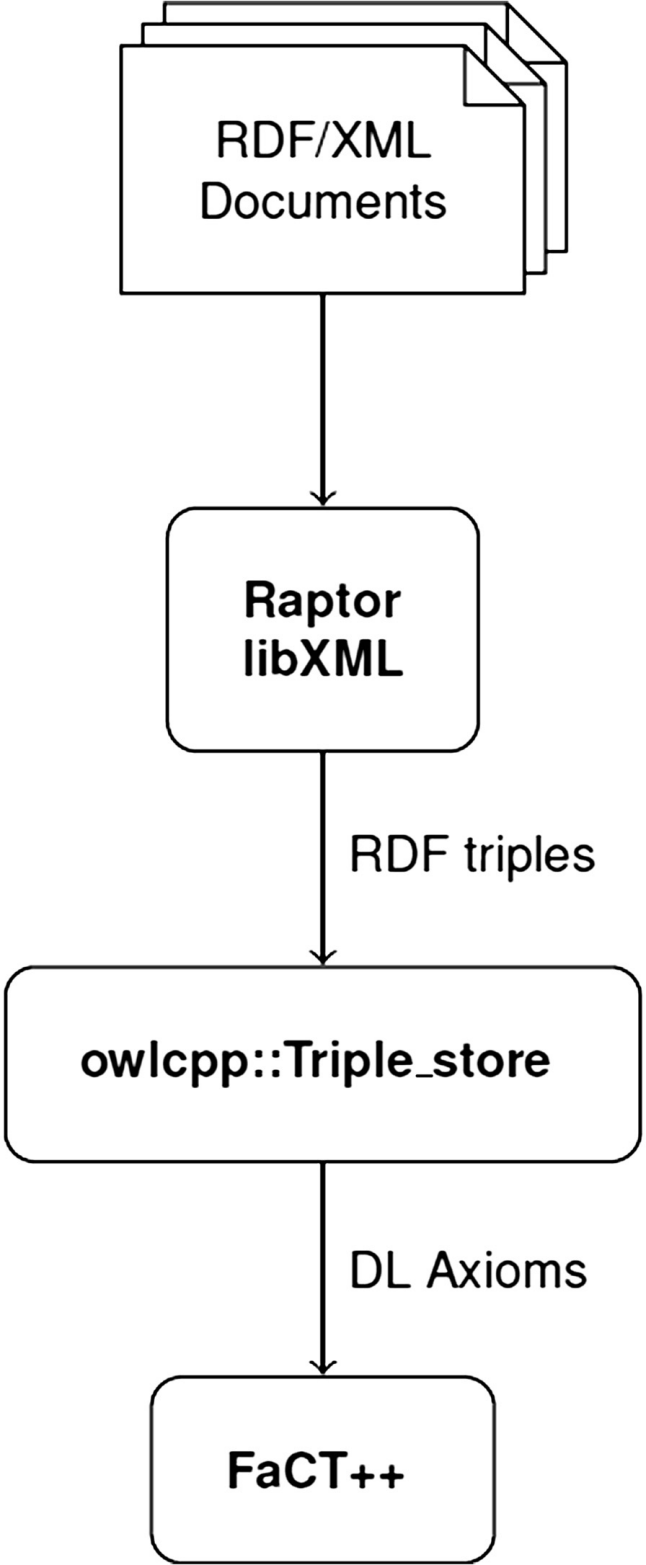
Workflow of the *owlcpp* library. *Owlcpp* loads RDF/XML documents from the file system, parses them using the Raptor RDF Syntax library, stores RDF triples in a triple store, converts the triples to OWL axioms, and passes these to FACT++ for reasoning

We sought to fill this gap by developing a library with the following key features: (i) supports fast loading and searching of large ontologies, (ii) has a small memory footprint, (iii) provides cross-platform compatibility, and (iv) can be accessed from multiple programming languages. The resulting library, *owlcpp*, is designed to support a common workflow where OWL ontologies written in RDF/XML are loaded from the file system and submitted to a reasoner for processing (Fig. 1). *owlcpp* is implemented in standard C++ and is aimed primarily at C++ and Python software developers. Here we discuss major design features and describe the results of an evaluation in which we compared the loading time, query time, and memory footprint of *owlcpp* and several other libraries across a set of ontologies of varying size and composition (Table 1).

**Table 1:** Ontologies used for evaluating *owlcpp*

Name	Size	Terms			Triples	Axioms
	MB	IRI	Literal	Blank		
OBP [29]	3.0	935	2366	5056	25,924	6109
OBI [30]	6.1	4106	10,115	8709	75,666	32,800
Uberon [31]	62.0	32,078	118,935	87,713	579,388	56,956
OpenGALEN part^a^	130.5	45,404	8423	771,980	2,004,170	187,893
VTO [37]	149.2	110,418	502,521	103,801	1,358,341	829,796
MESH [38]	193.8	916,056	249,740	4345	1,667,128	1,654,092
DRON [39]^b^	214.8	344,403	322,903	322,902	2,281,817	1,313,110
Biomodels [40]	253.2	232,214	535,725	481,909	2,686,610	1,905,822
OpenGALEN [41]	546.3	127,042	56,469	3,508,389	8,724,486	555,740

^a^OpenGALEN8_DD_2_Chapters.owl and its imports

^b^dron- ndc.owl

## Implementation

The design choices in implementing *owlcpp* were based on providing the four key features listed above. Specifically, *owlcpp* is developed in ISO-compliant C++03 [19], which ensures source-level portability, supports generation of language bindings, facilitates creation of concise and expressive APIs [20], and compiles into efficient executables. The API for *owlcpp* was designed to be concise without sacrificing usability and performance. In the interest of clarity and thread-safety, class methods and function arguments were declared const wherever possible.

The compatibility of *owlcpp* with different platforms was verified by compiling the library and executing the unit tests on the following platforms and compilers: Linux, Ubuntu 14.04 64-bit (gcc v4.8, Clang 3.5); Windows 7 64-bit (Microsoft Visual C++ 13); Mac OS X 10.6.8 (i686-apple-darwin10-gcc 4.2.1); Windows XP 32-bit (Microsoft Visual C++ 9, MinGW gcc 4.5.2).

Currently, *owlcpp* comprises three modules, *rdf*, for storing and searching RDF terms and triples; *io*, for loading ontology documents; and *logic*, for converting triples into axioms and passing them to a reasoner. The *io* module depends on the Redland Raptor [21], libXML2 [22], and iconv [23] libraries, and the *logic* module depends on FaCT++ [24, 25]. The *io* and *logic* modules have different external dependencies and can be built and used separately from each other. *owlcpp* also uses many of the Boost libraries, e.g., iterator, multi-index, and file system [26].

The basic features of *owlcpp*, as well as those of other key libraries, are shown in Table 2.

**Table 2:** Basic features of *owlcpp* and other similar libraries

Feature	*owlcpp*	Redland	Jena	OWL API
Load RDF/XML	✓	✓	✓	✓
Serialize RDF/XML	–	✓	✓	✓
Turtle I/O	–	✓	✓	✓
OWL/XML, Functional, Manchester I/O	–	–	–	✓
Search RDF triples	✓	✓	✓	–
Convert RDF to axioms	✓	–	✓	✓
Access FaCT++ reasoner	✓	–	–	✓
Access to other reasoners (Chainsaw, JFact, HermiT, Pellet, RacerPro)	–	–	–	✓
Axiom API	–	–	–	✓
C/C++ API	✓	✓	–	–
Python API	✓	✓	–	–
Java API	–		✓	✓

### *rdf* module

**Fig. 2:**
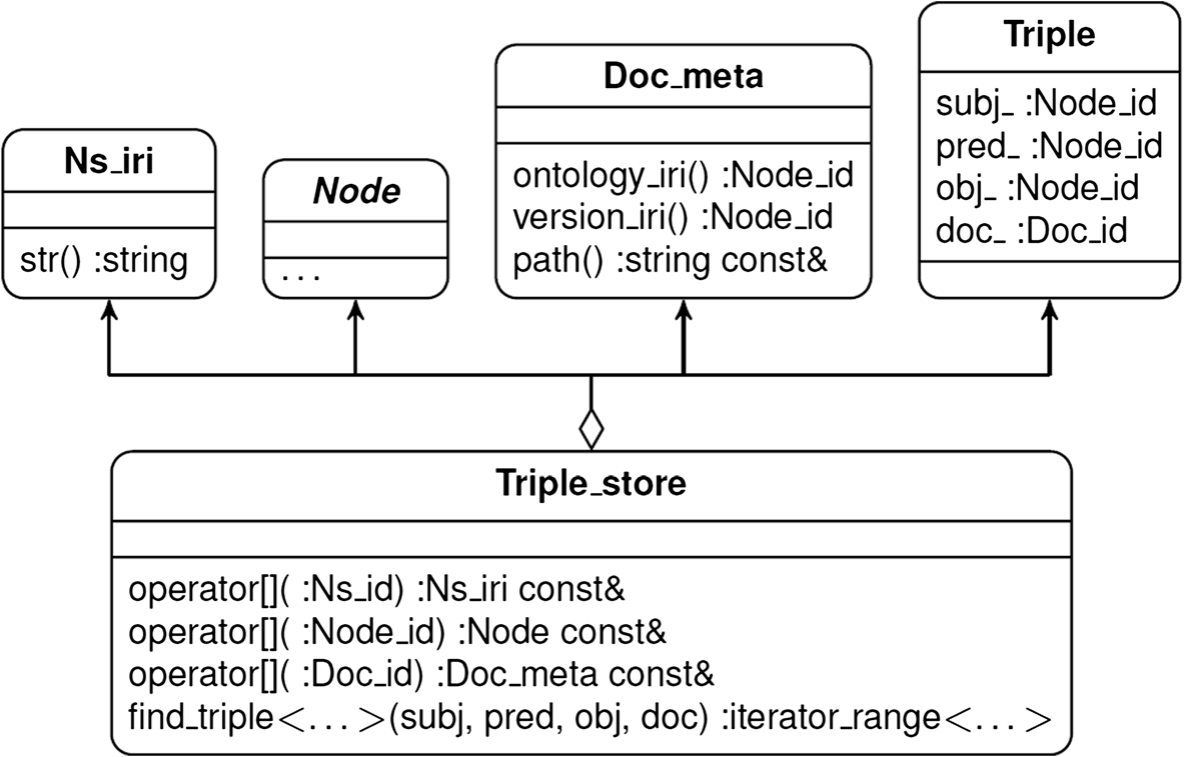
A diagram of the *owlcpp* Triple_store class, objects it stores, and some implemented methods. The Triple_store class serves as a container for prefix IRIs (Ns_iri), different types of RDF terms, which are accessed through the abstract Node interface, ontology document descriptions (Doc_meta), and RDF triples (Triple). Each object can be retrieved through an overloaded square bracket operator by supplying the object’s appropriately typed ID

The *rdf* module implements classes and methods as needed to support the RDF standard. To accommodate the demands of working with large ontologies, the design priorities for the module were compact in-memory representation of RDF terms and triples, and their efficient search and retrieval. The Triple_store class is the main container provided by the module. It supports storing, retrieving, and searching for prefix IRIs, RDF terms, ontology document descriptions, and RDF triples (Fig. 2). The library uses light-weight IDs to point to prefix IRIs (Ns_id), terms (Node_id), and document descriptions (Doc_id). The IDs for prefixes and terms standardized by RDF and OWL are defined by the library as compile-time constants.

**Fig. 3:**
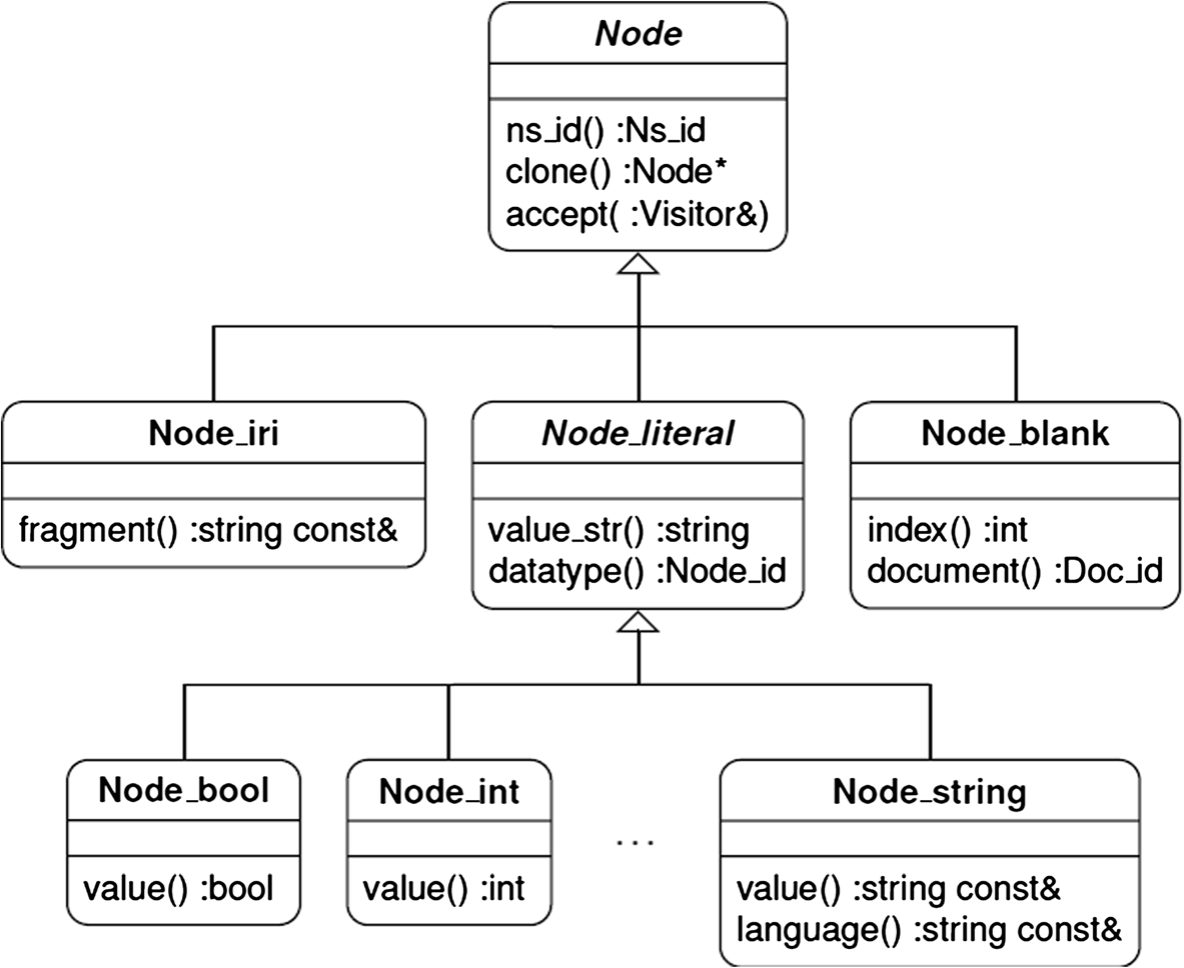
The RDF term type hierarchy as implemented in *owlcpp*. The RDF standard defines three types of terms implemented in *owlcpp* as the Node_iri, Node_literal, and Node_blank classes, which are accessed through the abstract Node interface. The abstract Node_literal class is further subtyped by concrete literal classes that represent the literal values as appropriate internal types, e.g., Node_bool for boolean types, Node_int for integers, etc. String-valued literals may also specify the language of their string values

The RDF standard defines three types of terms implemented in *owlcpp* as the Node_iri, Node_literal, and Node_blank classes (Fig. 3) [4]. These are accessed through the interface defined by the abstract Node class. A Node_iri object represents a prefix IRI with an optional fragment identifier. A Node_literal object stores an ID pointing to a Node_iri defining the datatype (e.g., xsd:date, rdf:PlainLiteral), a value as an appropriate internal type defined by subclasses of Node_literal, and, in the case of a string-valued literal, a language. A Node_blank object stores the ID of the document in which it was defined and an integer uniquely identifying the blank node within the document. In this way, blank nodes from different documents added to the same triple store are always kept distinct.

The ontology document descriptions (Doc_meta class) store Node_id’s for the ontology and version IRIs and for the file system path for the ontology document.

The Triple class represents RDF triples by defining a combination of subject, predicate, and object terms. Since each term may appear in many triples, the Triple class stores a light-weight ID rather than a value for each of the terms. In addition, Triple stores the ID of the source document.

Since term IDs do not distinguish between different types of terms, the Triple class cannot, by itself, enforce the type restrictions on its terms. For example, it is possible to create a triple where the subject term ID refers to a literal or where the predicate term ID refers to a blank node. While such triples will not be created during normal ontology parsing, it should still be noted that the Triple class implements a generalized RDF triple [4].

Searching stored triples is a frequent, performance-sensitive operation. The types of searches are application dependent and may involve matching any combination of subject, predicate, object, and document, while leaving other elements unspecified. To efficiently perform the required types of searches, *owlcpp* stores triples in several indices. Within an index, triples are separated into bins according to one term and sorted within each bin according to the other terms. For example, an index in which triples are binned by subject and sorted by predicate, object, and document can help to efficiently identify the triples matching a subject and a predicate. On the other hand, if matching an object and a predicate is required, an index configured to bin by object and sort by predicate, subject, and document is expected to perform better.

Since the indices have a significant memory footprint, the user can define the number and type of indices during compilation. By default, *owlcpp* uses two indices: (i) bin by subject and sort by predicate, object, and document, and (ii) bin by object and sort by predicate, subject, and document. These defaults were selected because they were empirically found to perform best for axiom generation, as described below.

Searching stored RDF triples is done using the find_triple method provided by Triple_store. The method is designed to automatically select the optimal search procedure based on the type of query and the available triple indices. The results of the search are returned as an iterator range [27], which is both efficient and convenient, because the range can be used to test the success of the search, to obtain the first match, or to iterate over all matching triples.

### *io* module

The *io* module provides several methods for loading RDF/XML ontology documents to a triple store. The documents can be loaded from the C++ Standard Library input streams using method load or directly from the filesystem using load_file. Loading an ontology document involves reading data from a stream, parsing XML structures, interpreting them as RDF statements, converting the statements into RDF terms and triples, and inserting them into a triple store. Currently, the Redland Raptor library is used as an RDF parser [16], which, in turn, relies on libxml [22] for parsing XML and iconv [23] for character encoding support.

The *io* module is designed for early detection of and recovery from errors. The errors may originate at different levels of the document loading process, such as opening a file or parsing XML, RDF, or OWL. An error occurring for any reason during document loading aborts the process by throwing an exception containing detailed information about its causes. In addition, instead of producing a triple store that contains an uncertain number of triples from the document that caused the error, the exception leaves the triple store in a valid state with none of the document’s content in the triple store. This behavior allows the user to utilize the existing triple store content or to attempt loading the document again.

OWL documents often import the contents of other documents, identifying them by their ontology IRIs and version IRIs. The *io* module provides a mechanism to automatically load the imports from the file system. To be able to locate the documents, the module implements a Catalog class that stores document descriptions and maps the document, ontology and version IRIs to file system paths. The module also provides a method for scanning file system directories for ontology documents and adding their metadata to the catalog. Supplying a Catalog object to the load or load_file methods causes the module to also load the imported documents to the triple store. Automatically loading documents from the Internet is not currently supported by the module because this feature introduces a significant uncertainty to the success and performance of ontology loading and adds complex operating system-specific dependencies to the library.

### *logic* module

The *logic* module is responsible for translating RDF triples into OWL axioms and facilitating interaction with reasoners. Translation of triples to axioms is implemented by following the W3C Recommendation [28]. If the triples do not meet some of the stated requirements, the process is aborted by throwing an exception containing detailed information about its causes. Axioms can be generated from the entire store, or from a subset of the triples. Generating an axiom associated with a particular triple usually requires information stored in other triples, which are found by searching the triple store. The axiom generation algorithm searches the triple store by subject, by subject and predicate, and by predicate and object.

Frequent search operations make configuration of triple indices an important factor affecting axiom generation performance. The optimal configuration was identified empirically by comparing axiom generation times using eight hand-picked index configurations and three different ontologies: the Ontology of Biological Pathways (OBP) [29], the Ontology for Biomedical Investigations (OBI) [30], and the Integrated Cross-species Anatomy Ontology (Uberon) [31]. For Uberon, the largest ontology of the three, selecting the best index configuration reduced the axiom generation time by a factor of 2.5 thousand.

Currently, the *logic* module works with FaCT++, which is, to our knowledge, the only open-source C/C++ reasoner library for OWL DL [24]. Logical queries are currently performed directly through the FaCT++ interface.

### Concurrency

Although *owlcpp* does not provide explicit support for concurrency, similar to C++ Standard Library containers, it is designed to maximize the number of thread-safe operations without penalizing performance. Operations that do not change the state of *owlcpp* containers (e.g., all const methods) are guaranteed to be thread-safe. On the other hand, if multiple threads concurrently access an *owlcpp* container object and at least one of the threads modifies its state (e.g., inserts an RDF triple), the behavior is undefined. Therefore the user is expected to ensure that a modifying thread obtains exclusive access to *owlcpp* containers.

### Build system

The build system for *owlcpp* is based on Boost.Build and is compatible with both Unix-like and Windows platforms [32, 33]. It is responsible for compilation and linking of static and shared variants of the library, as well as the sample executables according to the configuration provided by the user. The system also builds the required third-party dependencies from their sources, generates a Distutils module of *owlcpp* Python bindings, and produces API documentation using Doxygen [34].

### Unit tests

Unit tests comprise approximately 20 % of the library source code and cover most of its functionality. A separate test suite is implemented for each of the *owlcpp* modules. The tests for the *io* and *logic* modules make use of many small sample ontology documents that are part of the project source tree. Some of the documents are designed to test error detection at the XML, RDF, or OWL level. Some of the sample documents used by the *logic* module tests were adapted from the OWL 2 Test Cases [35]. The tests verify expected consistency of the ontologies and perform more specific logical queries. All unit tests are executed by the build system with a single command.

### Python bindings

The current version of *owlcpp* includes bindings for Python developed using the Boost.Python library. The functionality of the bindings is verified by a separate unit test suite. The bindings and their dependencies are packaged by the build system into a distributable Python module. APIs for other programming languages can also be exposed from *owlcpp* relatively easily and with minimal overhead. In future versions of *owlcpp*, these will be provided with the help of the SWIG library [36].

## Results

### Evaluation

To evaluate *owlcpp*, we compared the document loading time, triple query time, and memory foot print of *owlcpp* with those of Redland, Jena, and OWL API. Evaluation was conducted using five ontologies of varying size and composition: the Vertebrate Taxonomy Ontology (VTO) [37], Medical Subject Headings ontology (MESH) [38], Drug Ontology (DRON) [39], Biomodels Ontology [40], and OpenGALEN [41]. In addition, a one quarter portion of OpenGALEN (OpenGALEN part) was used. The statistics of the ontologies, including filesystem footprints and the counts for RDF terms, triples, and axioms are listed in Table 1.

To test the ability of the library to process large ontologies on off-the-shelf hardware, the tests were conducted on an underpowered, by current standards, computer. Performance was tested on a laptop with an Intel Core2 Duo T7700 2.40GHz CPU, 3GB of RAM, running Linux Ubuntu 14.04 64-bit. The performance of Java libraries was tested using Oracle Java JDK v1.7.0_51 with a 2 GB maximum memory pool (-Xmx2048m). Unless noted otherwise, *owlcpp* was compiled with gcc v4.8 using the default triple index configuration. Redland v1.0.13 with a hashed in-memory store, Jena v2.12.1 with an in-memory RDF store, and OWL API v4.0.1 were used for comparison, also with their default settings. The source code for performance tests can be found in Additional file 1. All testing was done using *owlcpp* v0.3.5.

### Ontology loading performance

**Fig. 4:**
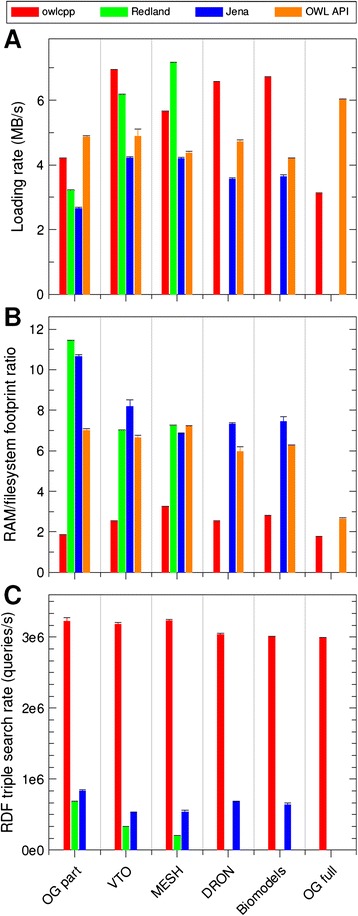
Performance comparison of the *owlcpp*, Redland, Jena, and OWL API libraries. The measurements were done using the following ontologies (by size, see Table 1): part of OpenGALEN (OG part), VTO, MESH, DRON, Biomodels, and complete OpenGALEN (OG full). The *bars* showing performance measurements are color-coded by library and grouped by ontology. The standard deviations of the measurements are shown as *error bars*. The corresponding bars are not shown if ontology loading failed. **a** shows ontology loading rates—ontology size divided by loading time. **b** shows the RAM footprint of each library after ontology loading normalized by the filesystem size of the ontology. **c** shows triple store querying rates. Each query identifies all triples matching a combination of a random subject and a constant predicate. Triple querying rates for OWL API are not shown because this operation is not supported

To evaluate ontology loading performance, each of the six ontologies was loaded into each of the four libraries. Some libraries were unable to load the larger ontologies. Jena was unable to load the complete OpenGALEN ontology, while Redland failed to load the complete OpenGALEN, Biomodels, and DRON ontologies. An attempt to load the complete OpenGALEN into Redland on a system with 32GB of RAM was also unsuccessful. The loading rates (size of the ontology file system footprint divided by the recorded loading time) is shown in Fig. 4a. The ontology loading rate of *owlcpp* ranges from 3.1 to 6.9 MB/s, while the range for the other libraries is from 2.6 to 7.2 MB/s. The *owlcpp* loading rate is faster than that of Jena for the five ontologies Jena could load and faster than that of Redland for two of the three ontologies Redland could load. Redland had a faster loading rate than *owlcpp* for MeSH. In addition, the *owlcpp* loading rate is faster than that of OWL API for four of the six test ontologies. OWL API has a faster loading rate for both OpenGALEN full and part.

### Memory footprint

The amount of memory required by each library during ontology loading was estimated by probing the resident set size of the process virtual memory. The peak RAM utilization normalized by the size of the ontology on the file system is shown in Fig. 4b. Of the libraries tested, *owlcpp* had the smallest memory footprint for all ontologies ranging from 1.8 to 3.2 bytes of RAM required for each byte on the file system. The same ratio ranged from 7.0 to 11.5 for Redland, from 6.8 to 10.7 for Jena, and from 2.6 to 7.0 for OWL API.

### Triple search efficiency

Searching by subject and predicate is the most common triples search during axiom generation. Therefore, the triple search performance of the libraries was tested by repeating queries where the subject was selected at random, and the predicate was rdfs:subClassOf. For each query, all matching triples were identified and counted. The number of queries for each test was selected so as to keep the test time at about one minute. The number of queries performed by each library divided by the elapsed time is shown on Fig. 4c. The *owlcpp* library showed significantly higher search rates ranging from 3.0 to 3.2 million queries per second (MQ/s). Jena showed significantly lower rates from 0.53 to 0.83 MQ/s. The rates for Redland ranged from 0.2 to 0.69 MQ/s. Note that OWL API was not included in this evaluation because it stores axioms rather than triples.

### Accuracy and error detection

In addition to evaluating the performance of *owlcpp*, we wanted to assess the accuracy of parsing and axiom generation. This was done using the OWL 2 Test Cases [35], some of which are incorporated into *owlcpp* unit tests. Further testing was done during the development of the Ontology of Biological Pathways (OBP) [29] by executing queries formulated by domain experts and comparing the results with ones from Protégé running with either FaCT++ or the HermiT reasoner plug-in [42]. The results of the queries were always identical.

Strict error checking has proven to be an important feature of *owlcpp*, helping to avoid incorrect semantic interpretation of ontologies and facilitating their development. Examples of errors detected by *owlcpp* but ignored by OWL API and Jena are undeclared property and annotation predicates and misspelled standard OWL terms.

## Discussion

*owlcpp* is a C++ library providing support for storing and searching RDF terms and triples, for loading RDF/XML documents along with their imports into a triple store, for generating OWL axioms based on stored triples, and passing axioms to the FaCT++ reasoner. To the best of our knowledge, *owlcpp* is the first C++ library for working with OWL ontologies.

Our primary goal was to design a library for software developers that would scale well for working with large ontologies. To facilitate use by software developers, we designed *owlcpp* to have a concise and expressive C++ API and an efficient Python API. For example, loading an ontology file into an *owlcpp* triple store can be accomplished with just two lines of code, whereas the same operation through the Redland Raptor library API requires over a dozen lines [43]. The API for RDF triple store search is another example. In *owlcpp*, a single method, Triple_store::find_triple(), can be used to search for triples matching a specific subject, predicate, object, document, or any combination thereof. The search is performed without sacrificing performance by selecting the most suitable triple index at compile time. The result of the search, an iterator range, can be used transparently to determine whether the triple store contains a triple matching the specified condition, to retrieve the first matching triple, or to iterate over all matching triples. On the other hand, the triple stores of both Redland and Jena define over ten different methods for searching triples.

Of critical importance to the utility of *owlcpp* is ensuring its scalability for use with large ontologies. Thus, we designed *owlcpp* to have a compact, in-memory storage of RDF terms and triples, efficient indexing of stored triples, and no virtual machine requirement. The latter facilitates *owlcpp*’s deployment in HPC environments. To evaluate the scalability of *owlcpp*, we compared its memory footprint, ontology document loading time, and triple query time with those of Jena, Redland, and OWLAPI. We found that *owlcpp* has a smaller memory footprint than the other three libraries for all ontologies tested (Fig. 4b), and we find that the ontology document loading time is faster for *owlcpp* than the other libraries for all tests with two exceptions (Fig. 4a): Redland was faster loading MesH, and OWLAPI was faster loading OpenGALEN or a part of OpenGALEN. The lower performance of *owlcpp* with the MeSH ontology is probably due to this ontology’s low ratio of triples to IRIs. In MeSH, each IRI appears, on average, in 1.8 triples, whereas in other ontologies this ratio ranges from 6.6 to 69. This property of MeSH increases the relative cost of IRI parsing, while diminishing the benefit of utilizing IRI IDs. Ontology loading by *owlcpp* is slower for OpenGALEN, either full or part, than for the other ontologies. This is probably due to a 50 % greater number of triples per megabyte in the OpenGALEN ontology.

While interpreting the performance measurements of the *owlcpp*, Redland, Jena, and OWL API libraries, it is important to note significant differences in their architecture. *owlcpp* and Redland are natively-compiled libraries, whereas Jena and OWL API run under Java virtual machine and exhibit less deterministic performance and memory footprint due to just-in-time compilation and garbage collection. Furthermore, while *owlcpp*, Redland, and Jena store the documents in memory as a set of RDF triples, OWL API immediately converts the triples into axioms and annotations, which, arguably, can be stored in memory more compactly. Nevertheless, the performance comparison is useful because it helps predict the hardware requirements for a task and reflects on the overall user experience.

There are several limitations of *owlcpp*, which will be addressed in future versions. First, RDF/XML is the only OWL format currently supported by *owlcpp*. Future versions will introduce support for additional syntaxes, particularly Manchester, Turtle, and OWL/XML. Second, *owlcpp* doesn’t currently provide a Java API, and is therefore not interoperable with most of the currently available RDF/OWL tools. In future versions, we will provide a Java API. Third, although it is possible to manually add more nodes and triples to an *owlcpp* triple store, it is not currently possible to save the new RDF graph. Another important limitation of *owlcpp* is the lack of a description logic expression and axiom interface for axiom editing. Future versions will include this and will also improve readability of error messages, provide options for less strict parsing and axiom generation, and include a module for batch execution of OWL 2 Test Cases. Finally, future versions of *owlcpp* will provide an axiom-based in-memory data structure.

## Conclusions

*owlcpp* presents a number of benefits for developers and users. Its compact datamodel and efficient execution make it possible to work with large ontologies using off-the-shelf hardware. As a native library, *owlcpp* does not depend on a virtual machine installation, facilitating its deployment in HPC environments. The C++ and Python APIs of *owlcpp* are concise and expressive and facilitate its integration with other software modules. Currently, *owlcpp* is used in many groups to work with biological ontologies as well as in other fields including virtual reality, robotics, image analysis, and answer set programming.

## Availability and requirements

Project name: *owlcpp*

Project home page: http://owl-cpp.sourceforge.net/

Operating system(s): Cross-platform (tested: Linux, Windows, Mac)

Programming language: C++, Python

Other requirements: Boost, libxml2, iconv (under Windows), Raptor, FaCT++

License: Boost Software License–Version 1.0

Any restrictions to use by non-academics: none

## Additional file

Additional file 1:
**This file contains the code used for the performance comparison.** (PDF 49 kb)
