# Supplementary material for: owlcpp: a C++ library for working with OWL ontologies
Source: J Biomed Semantics. 2015 Sep 16;6:35. doi: 10.1186/s13326-015-0035-z (PMC4574266; doi:10.1186/s13326-015-0035-z)
Supplement: Additional file 1: — This file contains the code used for the performance comparison. (PDF 49 kb) [file 13326_2015_35_MOESM1_ESM.pdf]

1 Performance test source code

1.1 Triple store search 3, *owlcpp*

```
#include <iostream>
#include "boost/filesystem.hpp"
#include "boost/foreach.hpp"
#include "boost/chrono/chrono.hpp"
#include "boost/chrono/chrono_io.hpp"
#include "boost/random/mersenne_twister.hpp"
#include "boost/random/uniform_int_distribution.hpp"

#include "owlcpp/rdf/triple_store.hpp"
#include "owlcpp/rdf/query_node.hpp"
#include "owlcpp/io/input.hpp"
#include "owlcpp/detail/vector_set.hpp"
using owlcpp::Triple;
using owlcpp::Node_id;
using owlcpp::any;
namespace ot = owlcpp::terms;

#include "sample_files.hpp"

/**
Load 25 Open Galen files into owlcpp triple store, search triples, keep time
*******************************************************************************/
int main(int argc, char* argv[]) {
   const std::size_t files_to_load = 25;
   const std::size_t searches_to_run = 1L * 1000 * 1000 * 100/*0*/;
   typedef boost::chrono::high_resolution_clock clock_t;
   typedef clock_t::time_point time_t;
   typedef boost::chrono::duration<double, boost::ratio<1,1000*1000> > dur_t;
   owlcpp::Triple_store ts;

   std::vector<std::string> fnv = open_galen_file_list(argv[1]);

   for(std::size_t i = 0; i != fnv.size() && i != files_to_load; ++i) {
      load_file(fnv[i], ts);
   }

   owlcpp::Vector_set<Node_id> v;
   BOOST_FOREACH(Triple const& tr, ts.map_triple()) {
      v.insert(tr.subj_);
   }

   boost::mt11213b rg;
   boost::random::uniform_int_distribution<std::size_t> rd(0, v.size());
   const time_t t0 = clock_t::now();
   std::size_t n_results = 0;
   for( std::size_t n = 0; n != searches_to_run; ++n ) {
      const Node_id nid = v[rd(rg)];
      BOOST_FOREACH(
               Triple const& tr,
               ts.find_triple(nid, ot::rdf_type::id(), ot::owl_Class::id(), any())
      ) {
         ++n_results;
```

```
        }
    }

    const dur_t d0 = clock_t::now() - t0;
    std::cout
    << "triples: "<< ts.map_triple().size() << '\n'
    << "subject nodes: "<< v.size() << '\n'
    << "searches: " << searches_to_run << '\n'
    << "results: " << n_results << '\n'
    << "searching: " << d0 << '\n'
    << d0 / searches_to_run << " per search" << '\n'
    << std::endl;

    return 0;
}
```

1.2 Triple store search 3, Jena

```
package owl_java;
import java.io.File;
import java.io.InputStream;
import java.util.ArrayList;
import java.util.Iterator;
import java.util.List;
import com.hp.hpl.jena.rdf.model.Model;
import com.hp.hpl.jena.rdf.model.ModelFactory;
import com.hp.hpl.jena.rdf.model.RDFNode;
import com.hp.hpl.jena.rdf.model.ResIterator;
import com.hp.hpl.jena.rdf.model.Resource;
import com.hp.hpl.jena.rdf.model.StmtIterator;
import com.hp.hpl.jena.util.FileManager;
import com.hp.hpl.jena.vocabulary.RDFS;

/** Load some Open Galen files into Jena triple store, run queries keep time */
public class Jena_rdf_04 {

    public static void main(String[] args) {
        int files_to_load = 250;
        long searches_to_run = 200L * 1000 * 1000;
        List<File> l = Sample_data.files_OG(args[0]);
        Model model = ModelFactory.createDefaultModel();
        int nf = 0;
        for( Iterator<File> i = l.iterator(); i.hasNext() && nf < files_to_load; ++nf)
             {
            InputStream in = FileManager.get().open(i.next().toString());
            model.read( in, "");
        }

        ArrayList<Resource> rl = new ArrayList<>();
        for( ResIterator i = model.listSubjects(); i.hasNext(); ) {
            rl.add(i.next());
        }

        long t0 = System.currentTimeMillis();
        long n_results = 0;
        for( long n = 0; n != searches_to_run; ++n) {
            int ri = (int)(Math.random() * rl.size());
```

```
        Resource r = rl.get(ri);
        for(
              StmtIterator i = model.listStatements(r, RDFS.subClassOf, (RDFNode)
                 null);
              i.hasNext();
              i.next()
              ) {
           ++n_results;
        }
     }

     long t1 = System.currentTimeMillis();
     double dt = t1 - t0;
     System.out.println("triples: " + model.size());
     System.out.println("searches: " + searches_to_run);
     System.out.println("results: " + n_results);
     System.out.println("searching: " + dt / 1000.0);
     System.out.println( dt * 1000 / searches_to_run + " us per search");
  }
}
```

1.3 Triple store search 3, Redland

```
#include "redland.h"
#include "raptor2.h"
#include <iostream>
#include "boost/filesystem.hpp"
#include "boost/chrono/chrono.hpp"
#include "boost/chrono/chrono_io.hpp"
#include "boost/random/mersenne_twister.hpp"
#include "boost/random/uniform_int_distribution.hpp"

#include "owlcpp/detail/vector_set.hpp"
#include "sample_files.hpp"

struct Node_compare{
   bool operator()(librdf_node const* n1, librdf_node const* n2) const {
      return raptor_term_compare(n1, n2) < 0;
   }
};

/**
Load some Open Galen files into Redland triples store, run queries, keep time
*******************************************************************************/
int main(int argc, char* argv[]) {
   const std::size_t files_to_load = 25;
   const std::size_t searches_to_run = 20 * 1000 * 1000;
   librdf_world* world = librdf_new_world();
   librdf_world_open(world);
   librdf_storage* storage = librdf_new_storage(world, "hashes", NULL, "hash-type='
      memory'");
   librdf_model* model=librdf_new_model(world, storage, NULL);
   librdf_parser* parser = librdf_new_parser(world, "rdfxml", NULL, NULL);
   typedef boost::chrono::high_resolution_clock clock_t;
   typedef clock_t::time_point time_t;
   typedef boost::chrono::duration<double, boost::ratio<1,1000*1000> > dur_t;
```

```
std::vector<std::string> fnv = open_galen_file_list(argv[1]);

std::cout << "loading .. ";
std::cout.flush();
for(std::size_t i = 0; i != fnv.size() && i != files_to_load; ++i) {
   librdf_uri* uri = librdf_new_uri_from_filename(world, fnv[i].c_str());
   librdf_parser_parse_into_model(parser, uri, NULL, model);
   librdf_free_uri(uri);
}
librdf_free_parser(parser);
std::cout << "done" << std::endl;

std::cout << "finding subject nodes .. ";
std::cout.flush();
owlcpp::Vector_set<librdf_node*, Node_compare> v;
librdf_stream* stream = librdf_storage_serialise(storage);
for( ; ! librdf_stream_end(stream); librdf_stream_next(stream) ) {
   librdf_statement* statement = librdf_stream_get_object(stream);
   librdf_node* node = librdf_statement_get_subject(statement);
   if( v.find(node) == v.end() ) v.insert(librdf_new_node_from_node(node));
}
librdf_free_stream(stream);
std::cout << "done" << std::endl;

unsigned char const* rdf_type_str =
         (unsigned char const*)"http://www.w3.org/1999/02/22-rdf-syntax-ns#type";
librdf_node* rdf_type = librdf_new_node_from_uri_string(world, rdf_type_str);
unsigned char const* owl_Class_str =
         (unsigned char const*)"http://www.w3.org/2002/07/owl#Class";
librdf_node* owl_Class = librdf_new_node_from_uri_string(world, owl_Class_str);
unsigned char const* rdfs_subClassOf_str =
         (unsigned char const*)"http://www.w3.org/2000/01/rdf-schema#subClassOf";
librdf_node* rdfs_subClassOf = librdf_new_node_from_uri_string(world,
   rdfs_subClassOf_str);

boost::mt11213b rg;
boost::random::uniform_int_distribution<std::size_t> rd(0, v.size());
const time_t t0 = clock_t::now();
std::size_t n_results = 0;

for( std::size_t n_searches = 0; n_searches != searches_to_run; ++n_searches) {
   librdf_node* n = v[rd(rg)];
   librdf_iterator* i;
   for(
         i = librdf_storage_get_targets(storage, n, rdfs_subClassOf);
         ! librdf_iterator_end(i);
         librdf_iterator_next(i)
   ) {
      ++n_results;
   }
   librdf_free_iterator(i);
}

const dur_t d0 = clock_t::now() - t0;
std::cout
<< "triples: "<< librdf_model_size(model) << '\n'
<< "subject nodes: "<< v.size() << '\n'
```

```
    << "results: " << n_results << '\n'
    << "searching: " << d0 << '\n'
    << d0 / searches_to_run << " per search" << '\n'
    << std::endl;

    librdf_free_node(rdf_type);
    librdf_free_node(owl_Class);
    librdf_free_node(rdfs_subClassOf);
    BOOST_FOREACH(librdf_node* n, v) librdf_free_node(n);
    librdf_free_model(model);
    librdf_free_storage(storage);
    librdf_free_world(world);
    return 0;
}
```
